# Triptolide Prevents Bone Destruction in the Collagen-Induced Arthritis Model of Rheumatoid Arthritis by Targeting RANKL/RANK/OPG Signal Pathway

**DOI:** 10.1155/2013/626038

**Published:** 2013-03-12

**Authors:** Chunfang Liu, Yanqiong Zhang, Xiangying Kong, Liuluan Zhu, Jian Pang, Ying Xu, Weiheng Chen, Hongsheng Zhan, Aiping Lu, Na Lin

**Affiliations:** ^1^Department of Properties Theory of Traditional Chinese Medicine, Institute of Chinese Materia Medica, China Academy of Chinese Medical Sciences, No. 16, Nanxiaojie, Dongzhimennei, Beijing 100700, China; ^2^Institute of Infectious Diseases, Beijing Ditan Hospital, Capital Medical University, Beijing 100015, China; ^3^Shi's Center of Orthopedics and Traumatology, Shuguang Hospital Affiliated to Shanghai University of Traditional Chinese Medicine, Shanghai 201203, China; ^4^Wangjing Hospital, China Academy of Chinese Medical Sciences, Beijing 100700, China; ^5^Institute of Basic Research of Clinical Medicine, China Academy of Chinese Medical Sciences, Beijing 100700, China

## Abstract

Focal bone destruction within inflamed joints is the most specific hallmark of rheumatoid arthritis (RA). Our previous study indicated that the therapeutic efficiency of triptolide in RA may be due partially to its chondroprotective and anti-inflammatory effects. However, its roles in bone destruction are still unclear. In this study, our data firstly showed the therapeutic effects of triptolide on severity of arthritis and arthritis progression in collagen-induced arthritis (CIA) mice. Then, by micro-CT quantification, triptolide treatment significantly increased bone mineral density, bone volume fraction, and trabecular thickness and decreased trabecular separation of inflamed joints. Interestingly, triptolide treatment could prevent the bone destruction by reducing the number of osteoclasts in inflamed joints, reducing the expression of receptor activator of NF-**κ**B (RANK) ligand (RANKL) and RANK, increasing the expression of osteoprotegerin (OPG), at both mRNA and protein levels, and decreasing the ratio of RANKL to OPG in sera and inflamed joints of CIA mice, which were further confirmed in the coculture system of human fibroblast-like synovial and peripheral blood mononuclear cells. These findings offer the convincing evidence for the first time that triptolide may attenuate RA partially by preventing the bone destruction and inhibit osteoclast formation by regulating RANKL/RANK/OPG signal pathway.

## 1. Introduction

Rheumatoid arthritis (RA) represents a chronic autoimmune disease characterized by the presence of inflammatory synovitis, the predominance of proerosive mediators, and the progressive destruction of cartilage and bone [1]. Focal bone destruction within inflamed joints is the most specific hallmark of RA and leads to deformation, laxity, and functional disability [2]. Osteoclasts, the primary bone resorptive cells, play an important role in bone destruction [3]. It has been demonstrated that osteoclasts are mainly located in the synovial inflammatory tissue, and the bone destruction occurred in RA belongs to osteoclast-mediated bone destruction that is regulated by the receptor activator of nuclear factor-*κ*B (RANK) ligand (RANKL) [4]. Both RANK and its ligand RANKL are crucial regulators of osteoclast differentiation. Under the physiological condition, RANKL is expressed on osteoblasts and activated T cells [5]. It triggers osteoclast maturation and bone resorption by binding with its receptor RANK on osteoclasts. As a member of the tumor necrosis factor (TNF)-receptor family and a soluble decoy receptor for RANKL, osteoprotegerin (OPG) is normally expressed by osteoblasts and inhibits bone resorption by binding with RANKL, which subsequently prevents RANKL binding to RANK [6]. Under the pathological condition, RANKL/RANK/OPG signal pathway plays a crucial role in the process of bone destruction [7]. RANKL induces osteoclast-mediated bone destruction, and OPG protects against bone destruction by inhibiting the binding of RANKL with its receptor RANK [8]. Bone resorption is regulated locally by the relative levels of expression of RANKL and OPG. Thus, RANKL/RANK/OPG signal pathway has been considered as a potential target for preventing systemic joint destruction in RA patients.

In the current treatments for RA, there are three kinds of therapeutic agents: disease modifying antirheumatic drugs (DMARDs) such as methotrexate, leflunomide, hydroxychloroquine, and glucocorticoids; nonsteroidal anti-inflammatory drugs (NSAIDs) such as dichlorophen, loxoprofen, and nabumetone; steroid and biological response modifiers, which are all clinically used to relieve the severity of RA, slow this disease progression, and prevent the subsequent joint damage [9, 10]. However, the clinical use of these therapies has been limited because of their adverse effects with a high frequency and high cost of treatment. Extracts of the herb Tripterygium Wilfordii Hook f. (TWHF), also known as “Lei Gong Teng”, are clinically used as one of the most common systemic treatments for (auto) immune disorders including RA, immune complex nephritis, and systemic lupus erythematosus [11, 12]. It has been extensively used for centuries in China because of its favorable cost-benefit ratio. In 2009, Goldbach-Mansky et al. [13] performed clinical trials to validate the disease-modifying effects of extracts of TWHF on patients with RA. The treatment with this extract administered over 24 weeks may be both effective and safe in treating patients with active RA. The rapid improvement in function and pain and the profound effect on inflammation may make it an attractive and affordable alternative to currently available agents. As an active compound of TWHF, triptolide is immunosuppressive, cartilage protective, and anti-inflammatory *in vivo* and effective on both humans and animals inflicted by a range of inflammatory and autoimmune diseases, such as RA [14–16]. A large number of recent studies has found its mechanisms on the treatment of RA [17–19]. However, the roles of triptolide in bone destruction, which is a major problem of RA, are still unclear.

Collagen-induced arthritis (CIA) by the immunization of DAB/1 mice with type II collagen in complete Freund's adjuvant is widely used for the study of autoimmune arthritis [20]. This model exhibits joint swelling, synovitis, periosteal new bone formation, articular bone erosion, and osteopenia [21]. Because this model mimics many of the clinical and pathological features of human RA, we here investigated the protective effect of triptolide on bone destruction in CIA mice.

## 2. Materials and Methods

The study was approved by the Research Ethics Committee of Institute of Chinese Materia Medica, China Academy of Chinese Medical Sciences, Beijing, China. All animals were treated in accordance with the guidelines and regulations for the use and care of animals of the Center for Laboratory Animal Care, China Academy of Chinese Medical Sciences.

### 2.1. Animals

Seventy-two male DBA/1 mice (6~8 weeks old) were purchased from Charles River Laboratory Japan, Kanagawa, Japan. All mice were maintained in a room equipped with an air-filtering system, and the cages and water were sterilized.

### 2.2. Induction of CIA

CIA was induced as our previously reported study [22, 23]. Briefly, bovine type II collagen (Chondrex, Redmond, WA, USA) was dissolved in 0.1 M acetic acid overnight at 4°C. This was emulsified in an equal volume of complete Freund's adjuvant (Chondrex, Redmond, WA, USA). The mice were immunized intradermally at the base of the tail with 100 *μ*L of emulsion containing 100 *μ*g of type II collagen. On day 21, mice were boosted intraperitoneally with 100 *μ*g type II collagen dissolved in phosphate buffered saline (PBS).

### 2.3. Treatment and Groups

Triptolide (purity > 99.98%) was kindly provided by Professor Sui Lin (Fujian Institute of Medical Sciences, Fuzhou, China), and this is commercially available from Alexix Biochemicals (San Diego, CA, USA). Methotrexate was purchased from Sigma (St. Louis, MO, USA). Both were dissolved in 0.05% DMSO (Sigma, St. Louis, MO, USA). The routes of triptolide and methotrexate delivery were both oral administration. Treatment was given daily for a period of 21 days. The dosage selection for triptolide [8–32 *μ*g/(kg·day)] was according to our previous study [22] and corresponded to 0.625–2.5% of LD50 for triptolide (1.278 mg/kg).

Seventy-two DBA/1 mice were divided into 6 groups with the equal number (*n* = 12): normal control group (Normal), CIA model control group (Vehicle), CIA mice treated with 8 *μ*g/(kg·day) triptolide (Trip 8), 16 *μ*g/(kg·day) triptolide (Trip 16), 32 *μ*g/(kg·day) triptolide (Trip 32), and 0.1 mg/kg methotrexate (MTX).

### 2.4. Severity Assessment of Arthritis

Mice were observed once every 2-3 days after primary immunization. Arthritis severity was evaluated by arthritis index, arthritis incidence, and percentage of arthritic limbs which were performed by two independent, blinded observers. All four limbs of the mice were evaluated and scored from 0 to 4 according to the following scale: 0, normal; 1, detectable arthritis with erythema at least some digits; 2, significant swelling and redness; 3, severe swelling and redness from joint to digit; and 4, maximal swelling with ankylosis. The total score was the cumulative value for the four limbs, with a maximum of 16 for each mouse. Arthritis was considered to be present if the score for a paw was >2. The incidence of arthritis is defined as the percentage of animals within each group exhibiting any sign of disease regardless of severity. In addition, the number of arthritic limbs of individual mice were counted and added to represent the number of arthritic limbs in a group. The percentage of arthritic limbs in a group was calculated as the following formula:

(1) percentage of arthritic limbs =number of arthritic limbs in a groupnumber of all limbs in a group×100%.

### 2.5. Change in Body Weight after Onset of Arthritis

Because the loss of body mass is associated with aggressive progression of RA in clinics [24, 25], we detected the changes in body weight after the onset of arthritis in order to evaluate the side effect of triptolide in the treatment of RA. The change in body weight (%) of each individual CIA mice after the onset of arthritis was calculated as the following formula:

(2) change in body weight =(bodyweight(day 22 of arthritis)bodyweight(day 1 of arthritis)   −bodyweight(day 1 of arthritis)bodyweight(day 1 of arthritis))×100%.

### 2.6. Histopathology and TRAP Staining

Mice were sacrificed by cervical dislocation on day 22 of arthritis. The right hind limbs including the paws, ankles, and knees were dissected, fixed immediately for 2 h in 4% paraformaldehyde, decalcified in 10% EDTA for up to 1 month at 4°C, and embedded in paraffin. Tissue sections (5 *μ*m) were mounted on common slides for staining with hematoxylin and eosin. All sections were randomized and evaluated by two trained observers who were blinded to the treatment groups and the arthritis severity of each mouse. The data were expressed as mean bone destruction scores, which were based on a scale of 0–3, as our previous study [22]. Minor differences between observers were resolved by mutual agreement. To identify osteoclasts, sections were stained for tartrate-resistant acid phosphatase (TRAP) using an acid phosphatase kit (Sigma, St. Louis, MO, USA). TRAP-positive multinucleated cells that contained more than 3 nuclei were identified as osteoclasts, and these were counted by light microscopy.

### 2.7. Micro-CT Imaging

Three-dimensional reconstruction and sagittal images of the right knee and ankle joints were obtained by microfocal computed tomography (micro-CT, Explore Locus SP, GE, USA) at the day 22 of arthritis. Briefly, after the mice in different groups being killed using ether anesthesia, the hind limbs were removed and fixed in 4% paraformaldehyde for 2 h. The samples were scaned with micro-CT. Using the constructed sagittal image from micro-CT scanning data, histomorphometric analysis of the proximal tibial epiphysis exclude the cortical bone was performed in the measurement area facing the articular cavity from the begining of growth plate. Bone volume, bone mineral density (BMD), trabecular thickness, and trabecular separation at the tibial spongiosa were measured using an image analyzing system (Microview ABA2.2 software, GE, USA).

### 2.8. Osteoclast Formation in Coculture System of Synovial Fibroblasts and Peripheral Blood Mononuclear Cells

The coculture system of human fibroblast-like synovial cells (HFLS) and peripheral blood mononuclear cells (PBMCs) were constructed according to the protocol of Nakano et al. [26]. Briefly, HFLS, derived from RA patients after synovectomy, were purchased from Cell Applications (San Diego, CA, USA) and were routinely cultured in synoviocyte growth medium (Cell Applications). Blood was collected from healthy volunteers, and their PBMCs were isolated by centrifugation over Ficoll/Paque at 400 g for 30 minutes. After isolation, PBMCs (2 × 10^5^ cells/well) were resuspended in *α*-minimum essential medium containing 10% fetal calf serum (FCS) and 50 ng/mL of macrophage colony-stimulating factor (M-CSF, PeproTech Inc. Rocky Hill, NJ, USA) and then seeded in 48-well tissue culture plates (Costar, Corning Inc, Corning, NY, USA). Three days later, adherent cells were used for subsequent cocultures with HFLS, which were then added into 48-well tissue culture plates (2 × 10^4^ cells/well) with PBMCs and cocultured for 9 days in MEM containing 10% FCS, 50 ng/mL of M-CSF, 10^-7 ^M 1,25- dihydroxyvitamin D_3_ (1,25[OH]_2_D_3_), and triptolide (0.28, 2.8, and 28 nmol/L, resp.). After that, some dishes were used for TRAP staining as aforementioned in Section 2.6; TRAP-positive multinucleated cells that contained more than 3 nuclei were identified as osteoclasts, and these were counted by light microscopy. 

### 2.9. RNA Isolation and Real-Time PCR

The left hind paws (including the paw and ankle) were dissected from mice on day 22 of arthritis, snap-frozen in liquid nitrogen, ground into powder, and homogenized. This procedure was done under RNase-free conditions. The RNA isolation and real-time PCR assay were carried out following the protocol of our previous study [22, 23]. Briefly, total RNA was extracted with TRIzol reagent (Invitrogen, Carlsbad, CA, USA) from the tissue homogenates according to the manufacturer's instructions. The total RNA (1 *μ*g) was reverse transcribed to cDNA using the QuantiTect Reverse Transcription Kit (QIAGEN K.K., Tokyo, Japan) according to the instructions manual. The specific transcripts were quantified by quantitative real-time PCR using QuantiTect SYBR Green PCR Kit (QIAGEN K.K., Tokyo, Japan) and analyzed with ABI 7500 real-time PCR system (Applied Biosystems, USA). Gene-specific primers were used for RANKL (5′-CAG CAT CGC TCT GTT CCT GTA-3′ as forward and 5′-CTG CGT TTT CAT GGA GTC TCA-3′ as reverse), RANK (5′-GCC CAG TCT CAT CGT TCT GC-3′ as forward and 5′-GCA AGC ATC ATT GAC CCA ATT C-3′ as reverse), OPG (5′-ACC CAG AAA CTG GTC ATC AGC-3′ as forward and 5′-CTG CAA TAC ACA CAC TCA TCA CT-3′ as reverse), and *β*-actin (5′-GGC TGT ATT CCC CTC CAT CG-3′ as forward and 5′-CCA GTT GGT AAC AAT GCC ATG T-3′ as reverse). The mRNA levels of RANKL, RANK, and OPG were normalized to *β*-actin mRNA level. PCR was performed as 40 cycles at 94°C for 15 s, 55°C for 30 s, and 72°C for 30 s. The relative mRNA expression was calculated with comparative *C*_*T*_ method.

### 2.10. Immunohistochemical Staining

Paraffin sections (5 *μ*m) of tissue from the knee and ankle joints were mounted on poly-L-lysine-coated slides. Immunolocalizations of RANKL and OPG in the joints were carried out with commercial Polink-2 plus Polymer HRP Detection System For Goat Primary Antibody kits (Golden Bridge International Inc., Mukilteo, WA, USA) according to the manufacturer's instructions. The paraffin sections were dewaxed by routine method and incubated for 10 min with 3% H_2_O_2_. Each section was incubated with normal goat serum for 20 min at room temperature, and then with primary antibodies against mouse RANKL (goat polyclonal antibody, dilution 1 : 50, Santa Cruz Biotechnology, Inc., Santa Cruz, CA, USA) and OPG (goat polyclonal antibody, dilution 1 : 50, Santa Cruz Biotechnology, Inc., Santa Cruz, CA, USA), respectively, overnight at 4°C. After incubation with polymer helper for 20 min at 37°C, sections were reacted with poly-HRP anti-goat IgG for 20 min at 37°C. The sections were then stained with 3,3-diaminobenzidine (Sigma, St. Louis, MO, USA) and counterstained with hematoxylin. For the control staining, PBS was used instead of the primary antibodies.

Specimens were examined using a Leica image analyzer and analyzed by computer image analysis (Leica Microsystem Wetzlar Gmbh., Wetzlar, Germany) in a blinded manner. To localize and identify areas with positively stained cells, ten digital images per specimen of synovium from a knee or ankle joint were recorded, and quantitative analysis was performed according to the color cell separation. The results are expressed as the mean region of interest, representing the percentage of area covered with positively stained cells per image at a magnification of ×400.

### 2.11. Enzyme-Linked Immunosorbant Assay

Sera from the mice on day 22 of arthritis and conditioned media from HFLS cultured in the presence of triptolide (0.28, 2.8, and 28 nmol/L, resp.) for 3 d, 7 d, 10 d, 14 d, 17 d, and 21 d was obtained and stored at −80°C until use. The amounts of RANKL and OPG in serum and OPG in supertant of HFLS were detected by ELISA assay (R&D system, Minneapolis, MN, USA) according to the manufacturer's protocol, and absorbance was measured at 450 nm. All experiments were done in triplicate. 

### 2.12. Western Blot Analysis

Isolated PBMCs (3 × 10^6^ cells/well) or HFLS (10^5^ cells/well) were cultured in 6-well plates for 3 weeks in the presence of triptolide (0.28, 2.8, and 28 nmol/L, resp.). The cells were washed twice with PBS and treated with lysis buffer (10 mM HEPES-KOH [pH 7.8], 15 mM KCl, 2 Mm MgCl_2_, 1% Igepal, 2 *μ*g/mL leupeptin, and 1 mM phenylmethylsulfonyl fluoride). The protein was obstained to detect the levels of RNAKL in HFLS and RNAK in PBMCs by Western blot. The Western blot protocol and semiquantitative analysis were carried out following the protocol of our previous study [23]. The following antibodies were used: RANKL antibody (goat polyclonal antibody, dilution 1 : 200, Santa Cruz Biotechnology, Inc., Santa Cruz, CA, USA), RANK antibody (goat polyclonal antibody, dilution 1 : 200, Santa Cruz Biotechnology, Inc., Santa Cruz, CA, USA), and GAPDH antibody (internal control, rabbit polyclonal antibody, dilution 1 : 200, Santa Cruz Biotechnology, Inc., Santa Cruz, CA, USA). All experiments were done in triplicate. 

### 2.13. Statistical Analysis

The software of SPSS version 13.0 for Windows (SPSS Inc, Chicago, IL, USA) and SAS 9.1 (SAS Institute, Cary, NC) was used for statistical analysis. Continuous variables were expressed as $\;\overset{-}{X}\pm s$. Arthritis incidence and percentage of arthritic limbs were analyzed by a chi-square test. Arthritis index, pathological scores and number of TRAP positive cells were analyzed with nonparametric statistics (Kruskal-Wallis test). Other data were analyzed by one-way ANOVA followed by LSD test. Differences were considered statistically significant when *P* was less than 0.05.

## 3. Results

### 3.1. Effects of Triptolide on Severity of Arthritis and Arthritis Progression

**Figure 1:**
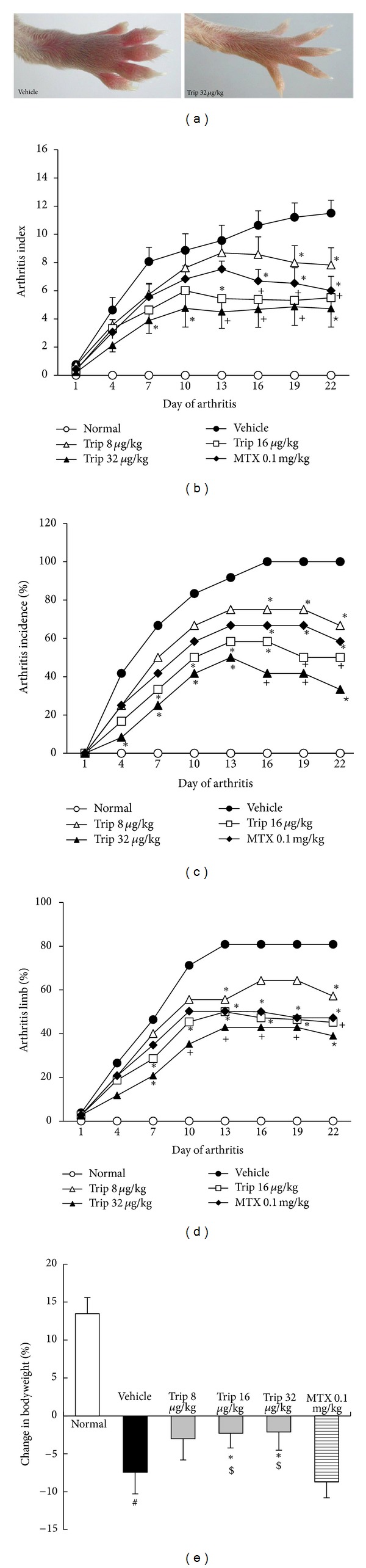
Effects of triptolide on severity of arthritis and arthritis progression in collagen-induced arthritis (CIA) mice. Mice were orally administered triptolide (Trip, 8, 16, and 32 *μ*g/kg, resp.), methotrexate (MTX, 0.1 mg/kg), or vehicle for 21 days from the first day of the onset of the clinical symptoms of arthritis. At the end of the experiment, the arthritis index, arthritis incidence, the percentage of arthritic limbs, and changes in bodyweight were evaluated. Data are represented as the mean ± SD (*n* = 12)., ^#^ *P* < 0.05 in comparison with the normal control. *P < 0.05,  ^+^ *P* < 0.01, and *P < 0.001 in comparison with the vehicle control. ^$^ *P* < 0.05 in comparison with methotrexate-treated CIA mice.

To investigate the effect of triptolide on arthritis, the CIA model in DBA/1 mice was used. Although the disease manifested itself on different days after immunization, we did not observe a relation between clinical response and time of onset of disease. *Consistent with the previous studies [22, 27], oral administration of triptolide, once a day started at the beginning of the fist rheumatoid arthritis signs such as erythema and/or oedema in joint(s) of CIA mice and continued for 21 days*. As shown in Figure 1(a), macroscopic evidence of arthritis such as erythema or swelling was markedly observed in vehicle-treated CIA mice, while a dose of 32 *μ*g/(kg·day) triptolide significantly attenuated arthritis severity in CIA mice. Additionally, the mean arthritis index (all *P* < 0.05, Figure 1(b)), arthritis incidence (all *P* < 0.05, Figure 1(c)), and the percentage of arthritis limbs (all *P* < 0.05, Figure 1(d)) in triptolide-treated mice were significantly lower than those in methotrexate-treated and vehicle-treated CIA mice with a dose-dependent manner. In the groups receiving triptolide with doses of 16~32 *μ*g/(kg·day), the rate of incidence and the percentage of arthritis limbs were markedly reduced from day 7 of arthritis. Remarkably, triptolide treatment could effectively suppress the loss of body weight of CIA mice (for Trip 16 group versus Vehicle: *P* < 0.05; for Trip 32 group versus Vehicle: *P* < 0.05; Figure 1(e)), but methotrexate treatment did not have this effect (Figure 1(e)). 

### 3.2. Triptolide Prevents Bone Destruction in CIA Mice

**Figure 2:**
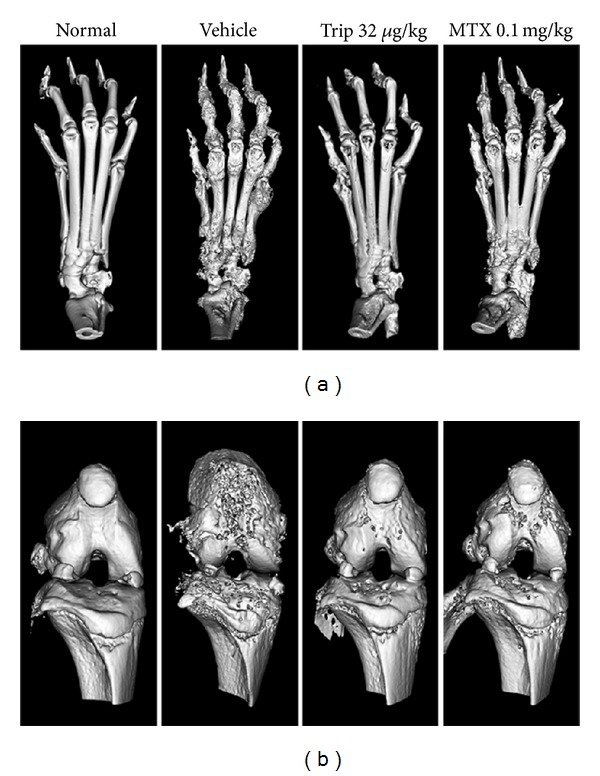
Micro-CT scan validates efficiency of triptolide in collagen-induced arthritis (CIA) mice. Mice were orally administered triptolide (Trip, 8, 16, and 32 *μ*g/kg, resp.), methotrexate (MTX, 0.1 mg/kg), or vehicle for 21 days from the first day of the onset of the clinical symptoms of arthritis. At the end of the experiment, Micro-CT scan was performed to validate the efficiency of triptolide in CIA mice. (a) and (b), respectively, showed the three-dimensional reconstructed bones of ankle and knee joints in different groups. Compared with vehicle-treated and methotrexate-treated CIA mice, dose of 32 *μ*g/(kg·day) triptolide markedly reduced the extent of joint destruction in triptolide-treated CIA mice.

**Figure 3:**
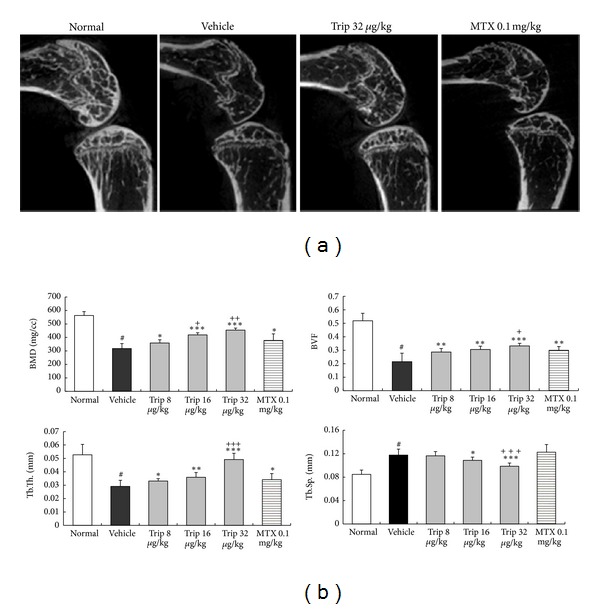
Inhibitory effects of triptolide in periarticular bone erosion in knee joints of collagen-induced arthritis (CIA) mice. Mice were orally administered triptolide (Trip, 8, 16, and 32 *μ*g/kg, resp.), methotrexate (MTX, 0.1 mg/kg), or vehicle for 21 days from the first day of the onset of the clinical symptoms of arthritis. At the end of the experiment, Micro-CT scan was performed to validate the efficiency of triptolide in CIA mice. (a) The two-dimensional reconstructed bones of knee joints in different groups. Compared with vehicle-treated CIA mice, dose of 32 *μ*g/(kg·day) triptolide markedly reduced the extent of joint destruction in triptolide-treated CIA mice. (b) The values of four parameters including bone volume, bone mineral density (BMD), trabecular thickness (Tb.Th), and trabecular separation (Tb.Sp) of proximal end of the tibia facing the articular cavity in different groups. Compared with vehicle-treated and methotrexate-treated CIA mice, triptolide significantly increased BMD, bone volume fraction (BVF), and Tb.Th of inflamed joints and decreased Tb.Sp of inflamed joints. Data are represented as the mean ± SD (*n* = 12). ^#^ *P* < 0.001 in comparison with the normal control. *P < 0.05,  **P < 0.01, and ***P < 0.001 in comparison with the vehicle control. ^+^ *P* < 0.05,  ^++^ *P* < 0.01, and ^+++^ *P* < 0.001 in comparison with methotrexate-treated CIA mice.

Micro-CT scan was performed to validate the efficiency of triptolide in CIA mice. Figures 2(a), 2(b), and 3(a), respectively, showed the three-dimensional reconstructed bones of knee joints in different groups. Compared with vehicle-treated and methotrexate-treated CIA mice, dose of 32 *μ*g/(kg·day) triptolide markedly reduced the extent of joint destruction in triptolide-treated CIA mice. In addition, four parameters including bone volume, BMD, trabecular thickness, and trabecular separation of inflamed joints in different groups were detected to quantify the extent of joint destruction. As shown in Figure 3(b), compared with vehicle-treated CIA mice, doses of 8~32 *μ*g/(kg·day) triptolide significantly increased BMD (all *P* < 0.05), bone volume fraction (all *P* < 0.01), and trabecular thickness of inflamed joints (all *P* < 0.05) and decreased trabecular separation (for Trip 16 group versus Vehicle: *P* < 0.05; for Trip 32 group versus Vehicle: *P* < 0.001), suggesting a protective role of triptolide on volume and quality of preserved trabecular bone despite joint inflammation. Notably, dose of 16 or 32 *μ*g/(kg·day) triptolide could also more effectively change these parameters than 0.1 mg/kg methotrexate could (all *P* < 0.05, Figure 3(b)).

**Figure 4:**
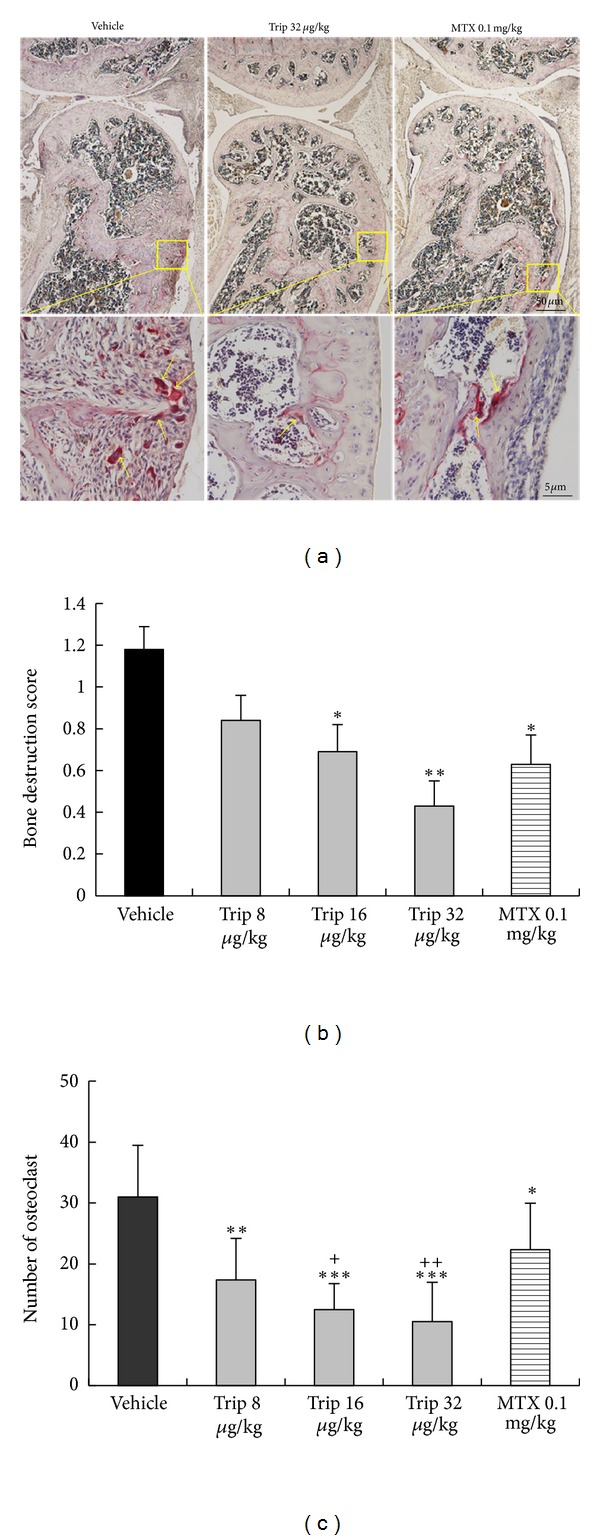
Triptolide inhibits osteoclast differentiation in CIA mice. Mice were orally administered triptolide (Trip, 8, 16, and 32 *μ*g/kg, resp.), methotrexate (MTX, 0.1 mg/kg), or vehicle for 21 days from the first day of the onset of the clinical symptoms of arthritis. At the end of the experiment, the osteoclast differentiation was evaluated. (a) Tartrate-resistant acid phosphatase (TRAP) stained section from knee joints of vehicle-treated, triptolide-treated, and methotrexate-treated CIA mice. The under graphs is the magnification of the yellow pane in the upper graphs, respectively. (b) The bone destruction score in knee joints of vehicle-treated, triptolide-treated, and methotrexate-treated CIA mice. (c) The number of osteoclasts (multinucleated TRAP positive cells) in knee joints of vehicle-treated, triptolide-treated, and methotrexate-treated CIA mice. Data are represented as the mean ± SD (*n* = 12). *P < 0.05,  **P < 0.01, and ***P < 0.001 in comparison with the vehicle control. ^+^ *P* < 0.05  and  ^++^ *P* < 0.01 in comparison with methotrexate-treated CIA mice.

Histopathological evaluation of knee joint sections of vehicle-treated CIA mice revealed inflammatory cell infiltration, synovial hyperplasia, and partial bone destruction. In contrast, oral administration of triptolide could distinctly reduce the extent of inflammatory cell infiltration and bone destruction (Figure 4(a)). To elucidate the effects of triptolide treatment on bone destruction at the histologic level, inflamed joints were scored with semiquantitative grading scales. As shown in Figure 4(b), the bone destruction scores in triptolide-treated CIA mice were significantly decreased with a dose-dependent tendency in comparison with vehicle-treated CIA mice (for Trip 16 group versus vehicle group: *P* < 0.05; for Trip 32 group versus vehicle group: *P* < 0.01). MTX also reduced significantly the bone destruction scores of inflamed joints compared with vehicle-treated CIA mice (*P* < 0.05, Figure 4(b)), although this value remained higher than those for triptolide-treated groups.

To confirm the effect of triptolide on the number of osteoclasts, knee joint sections were stained with TRAP. Only TRAP-positive multinucleated cells located at the bone surface within the bone destruction were considered as osteoclasts (Figure 4(a)). Compared with vehicle-treated CIA mice, the number of osteoclasts in the areas of bone destruction was significantly decreased in triptolide-treated mice with a dose-dependent tendency (all *P* < 0.01, Figure 4(c)). Methotrexate also reduced significantly the number of osteoclasts in the areas of bone destruction compared with vehicle control (*P* < 0.05, Figure 4(c)), although this value remained higher than those for triptolide-treated groups (all *P* < 0.001, Figure 4(c)).

### 3.3. Triptolide Inhibits Osteoclast Differentiation by Targeting RANKL/RANK/OPG Signal Pathway

**Figure 5:**
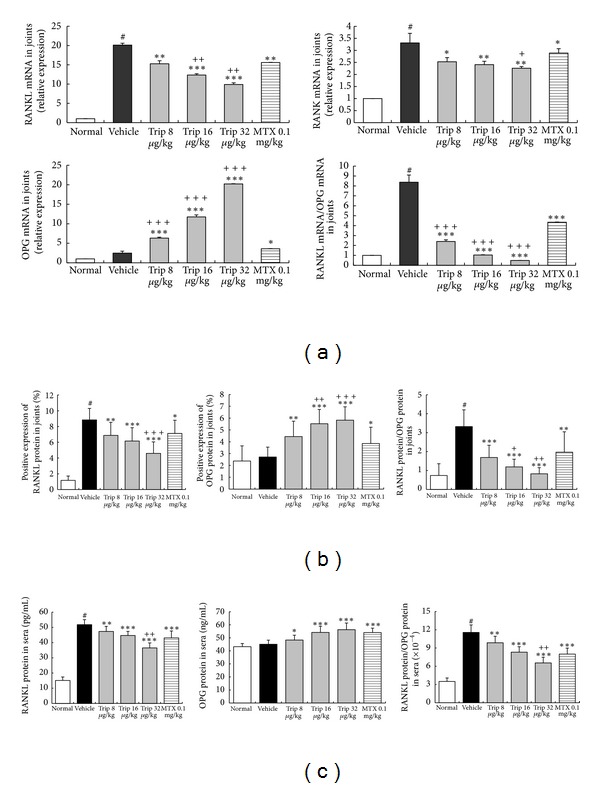
Triptolide inhibits osteoclast differentiation by targeting RANKL/RANK/OPG signal pathway. Mice were orally administered triptolide (Trip, 8, 16, and 32 *μ*g/kg, resp.), methotrexate (MTX, 0.1 mg/kg), or vehicle for 21 days from the first day of the onset of the clinical symptoms of arthritis. At the end of the experiment, the changes in RANKL, RANK, and OPG expression at both mRNA and protein levels in the ankle joint and serum were, respectively, detected by real-time PCR, immunohistochemistry, and ELISA assay. Triptolide reduces the expression of RANKL and RANK and enhances the expression of OPG and the ratio of RANKL to OPG in the ankle joint at mRNA (a) and protein (b) levels and in serum (c) of CIA mice. Data are represented as the mean ± SD (*n* = 12). ^#^ *P* < 0.001 in comparison with the normal control. *P < 0.05,  **P < 0.01, and ***P < 0.001 in comparison with the vehicle control. ^+^ *P* < 0.05,  ^++^ *P* < 0.01, and ^+++^ *P* < 0.001 in comparison with methotrexate-treated CIA mice.

To obtain insights into the mechanisms of the inhibitory effects of triptolide on bone destruction in inflamed joints of CIA mice, the expression of RANKL, RANK, and OPG at mRNA and protein levels in inflamed joints were, respectively, detected by quantitative real-time RT-PCR (Figure 5(a)) and immunohistochemistry (Figure 5(b)), and the serum levels of RANKL and OPG proteins were detected by ELISA assay (Figure 5(c)). Compared with vehicle-treated CIA mice, doses of 8~32 *μ*g/(kg·day) triptolide significantly reduced the expression of RANKL (all *P* < 0.001, only except for Trip 8 group at protein level in joints, Figure 5) and RANK (all *P* < 0.05, Figure 5(a)) and enhanced the expression of OPG (all *P* < 0.001, only except for Trip 8 group at protein level in joints and sera, Figure 5). More interestingly, triptolide treatments markedly reduced the ratio of RANKL to OPG in the sera and inflamed joints of CIA mice with a dose-dependent tendency (all *P* < 0.01, Figure 5). Methotrexate also reduced significantly the ratio of RANKL to OPG in the sera and inflamed joints of CIA mice compared with vehicle controls (all *P* < 0.01, Figure 5), although this value remained higher than those for triptolide-treated groups (*P* < 0.05, only except for Trip 8 group at protein level in joints, Figure 5).

**Figure 6:**
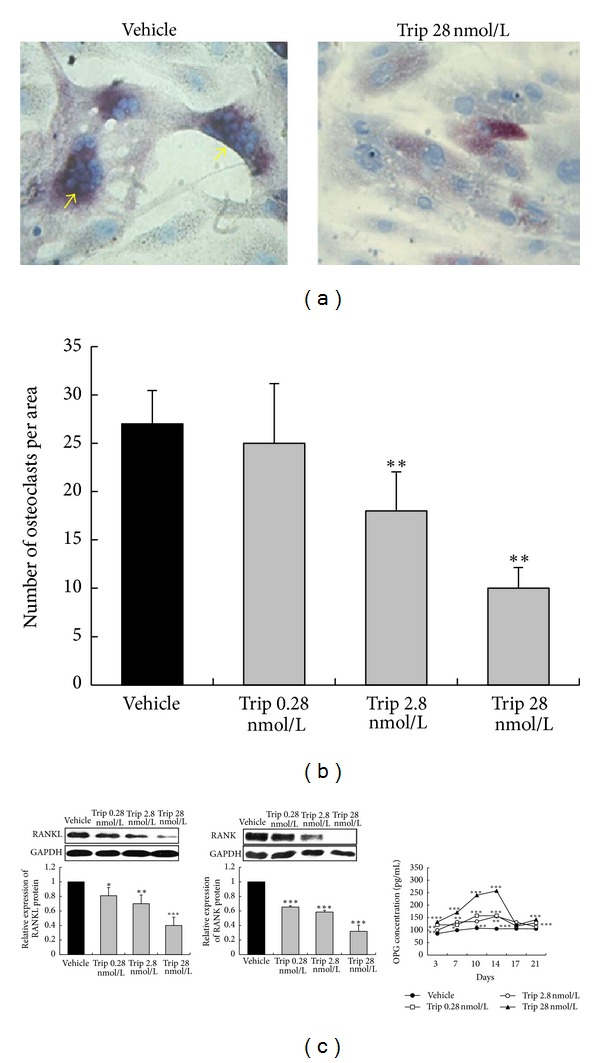
Triptolide inhibits osteoclastogenesis in the coculture system of human fibroblast-like synovial cells (HFLS) and peripheral blood mononuclear cells (PBMCs). (a) Numerous tartrate-resistant acid phosphatase- (TRAPs) positive multinucleated cells considered as osteoclasts were formed in the coculture system of HFLS and PBMCs, while few TRAP-positive multinucleated cells were formed when concentration of 28 nmol/L triptolide were added into this coculture system; (b) The number of osteoclasts per area counted under a light microscope; (c) Expression levels of RANKL and RANK proteins in PBMCs and HFLS measured by Western blot. Soluble OPG concentration in the culture supernatant of HFLS was measured by ELISA. Data are represented as the mean ± SD (*n* = 3). *P < 0.05,  **P < 0.01, and ***P < 0.001 in comparison with the vehicle control.

To further validate the above mechanisms, we also assessed the effects of triptolide on osteoclastogenesis in the coculture system of HFLS and PBMCs. Numerous TRAP-positive multinucleated cells considered as osteoclasts were formed (Figure 6(a)), but few TRAP-positive multinucleated cells were formed when concentration of 28 nmol/L triptolide were added into this coculture system (Figure 6(a)). The number of osteoclasts per area counted under a light microscope was significantly decreased in the coculture system with the addition of 2.8~28 nmol/L triptolide compared that without (*P* < 0.01, Figure 6(b)). Then, the soluble OPG concentration in HFLS measured by ELISA was increased and reached the peak at day 14 in the culture period (Figure 6(c)). Moreover, the expression level of RANKL protein in HFLS and that of RANK protein in PBMCs were also reduced significantly after the treatment of triptolide in a dose-dependent manner (all *P* < 0.05, Figure 6(c)). These results suggested that triptolide play a pivotal role in the osteoclastogenesis through the downregulation of RANKL and RANK, and the upregulation of OPG in the coculture system of HFLS and PBMCs.

## 4. Discussion

RA is a chronic and progressive inflammatory disease with multiple underlying pathogenic mechanisms caused by various risk factors. The disease progression of RA is associated with chronic soft tissue inflammation, which is often followed by bone and cartilage destruction of inflamed joints [28]. Therefore, to prevent bone and cartilage destruction is the most important issue in the treatment of RA. The therapeutic mechanisms of triptolide, a common medicine for RA therapy, have not been fully elucidated. Recent studies have mainly explained its suppressive effects on inflammation of RA. The previous study of our group has already demonstrated that triptolide can potently suppress the inflammatory responses and cartilage destruction in CIA mice [22]. Thus, this research is focus on the effects of triptolide on bone destruction in CIA mice. The main findings of our study are as the following two points: (1) triptolide attenuates RA partially by preventing the bone destruction; (2) triptolide inhibits osteoclast formation by regulating RANKL/RANK/OPG signal pathway.

In order to determine the therapeutic efficiency of triptolide for the treatment of RA, methotrexate which is the first-line therapy for this disease was used as the control drug. Methotrexate is not only effective against inflammatory symptoms but also in the prevention of bone destruction. However, accumulating reports have indicated that high dosage or the prolonged administration of low dosage of methotrexate may cause distinctive osteopenia by inhibiting the osteoblast activity and stimulating the osteoclast recruitment [29, 30]. Thus, it is of great clinical significance to find a novel drug which can effectively prevent both the joint damage and the systemic bone mass loss of RA patients. In line with the data of our previous study [22], we here first found that triptolide, as well as methotrexate, efficiently attenuated the severity of arthritis in CIA mice by reducing the mean arthritis index and the percentage of arthritic limbs, but without the subsequent loss of body weight. Then, triptolide improved histological findings in a dose-dependent manner by decreasing the extent of inflammatory cell infiltration and bone destruction in inflamed joints of CIA mice more effectively than methotrexate. These results were also confirmed by three-dimensional micro-CT, which showed that bone destruction and osteopenia were the common alterations of the bone in the affected joints in RA. Four parameters including bone volume, BMD, trabecular thickness, and trabecular separation of inflamed joints of three-dimensional micro-CT were detected to quantify these alterations in all CIA mice of different groups. Our data further suggest that oral administration of triptolide may effectively preserve both bone density and trabecular thickness of inflamed joints.

Recent studies have identified osteoclasts as the principal cell type responsible for bone destruction in RA [31, 32]. Thus, we hypothesized that triptolide would play a role in osteoclastogenesis in rheumatoid synovium. Consistent with this hypothesis, we found that the administration of triptolide significantly reduced the number of osteoclasts in the areas of bone destruction with a dose-dependent tendency by down-regulating the expression of RANKL and RANK, up-regulating the expression of OPG, and reducing the ratio of RANKL to OPG in CIA mice. Additionally, we also investigated the effects of triptolide on osteoclast formation in the coculture system of HFLS and PBMCs because HFLS can efficiently induce the formation of TRAP-positive multinucleated cells when cocultured with PBMCs [26, 33]. According to the results of *in vitro* study, we confirmed the inhibitive effects of triptolide on osteoclast formation which was found *in vivo* study.

In conclusion, our data offer the convincing evidence for the first time that triptolide may attenuate RA partially by preventing the bone destruction. Triptolide may inhibit osteoclast formation by regulating RANKL/RANK/OPG signal pathway, targeting which with triptolide may, therefore, be an important therapeutic strategy for preventing bone destruction in RA.
